# Diabetes mellitus and serum organochlorine pesticides mixtures in Mexican women

**DOI:** 10.1186/s12940-024-01096-w

**Published:** 2024-06-13

**Authors:** Rodrigo Ugalde-Resano, Ángel Mérida-Ortega, Belén Barajas, Lizbeth López-Carrillo, Mariano E. Cebrián

**Affiliations:** 1 Centro de Investigación en Salud Poblacional, Instituto Nacional de Salud Pública, Av. Universidad 655, Col. Santa María Ahuacatitlán, Cuernavaca, Morelos C.P. 62100 México; 2 Departamento de Toxicología, Centro de Investigación y de Estudios Avanzados del Instituto Politécnico Nacional, Av. Instituto Politécnico Nacional 2508, Col. San Pedro Zacatenco, México, C.P. 07360 México

**Keywords:** Organochlorine pesticides, Mixture analysis, Diabetes mellitus

## Abstract

**Background:**

Very recently, it has been reported that exposure to different mixtures of organochlorine pesticides (OCP) is associated with the development of diabetes mellitus (DM). In Mexico, DM is a public health problem that might be related to the historical intense use of OCP. We aimed to evaluate, the association between DM and serum concentrations of OCP mixtures, and identify the main contributors within them.

**Methods:**

We conducted a secondary cross-sectional analysis on the control group from a breast cancer population-based case-control study conducted from 2007 to 2011 in Northern Mexico. We identified 214 self-reported diabetic women and 694 non-diabetics. We obtained direct information about sociodemographic, lifestyle and reproductive characteristics. We determined 24 OCP and metabolites in serum by gas chromatography using an electron capture micro detector. We used Weighted Quantile Sum regression to assess the association of DM and exposure to multiple OCP, and the contribution of each compound within the mixture.

**Results:**

We found a positive adjusted association between DM and an OCP mixture (OR: 2.63, 95%CI: 1.85, 3.74), whose primary contribution arose from p, p’-DDE (mean weight 23.3%), HCB (mean weight 17.3%), trans nonachlor (mean weight 15.4%), o, p’-DDE (mean weight 7.3%), heptachlor epoxide (mean weight 5.9%), oxychlordane (mean weight 4.7%), and heptachlor (mean weight 4.5%). In addition, these OCP along with p, p’-DDT and cis chlordane, were of concern and remained associated when excluding hypertensive women from the analysis (OR 2.55; 95% CI 1.56, 4.18).

**Conclusions:**

Our results indicate, for the first time in a Latin-American population, that the concomitant exposure to multiple OCP is associated with DM. Further research is needed since the composition of OCP mixtures may vary according to regional pesticides use patterns.

## Introduction

Diabetes mellitus (DM) constitutes an enormous worldwide public health concern with around 22 and 460 million new and prevalent cases by 2019, respectively [1]. This disease is the main cause of blindness and kidney failure, with a substantial economic burden that has been calculated to represent 2.1% of the gross domestic product in United States [2, 3]. Until now, the etiology of DM is not fully understood, but overweight/obesity, age, family history, and possibly smoking have been identified as risk factors. Additionally, it has been suggested that this disease could have an environmental origin associated with potentially diabetogenic pollutants such as: arsenic, mercury, phthalates, bisphenol A, dioxins and organochlorine pesticides (OCP) [4].

The OCP are a diverse group of synthetic chemicals that have been widely used to control pests in food and cotton production, and vector-borne diseases and ectoparasites [5–7], however, their presence has been documented in recent human biological samples [8, 9]. Many countries have prohibited the production and/or application of various OCP due to their high toxicity and persistence in the environment, as well as their ability to volatilize, bioaccumulate and biomagnify in the food chain [5, 10].

Through one-by-one exposure approach, epidemiological evidence show DM is positively and consistently related to OCP [11]; particularly: dichlorodiphenyltrichloroethane (DDT) and/or its main metabolite dichlorodiphenyldichloroethylene (DDE) [8, 10, 12, 13]; heptachlor; hexachlorobenzene (HCB); dieldrin; chlordane; oxychlordane; transnonachlor; β-hexachlorocyclohexane (HCH); α-HCH, ϒ-HCH and endosulfan [8, 14–16]. To our knowledge, only few studies has associated DM with OCP exposure with mixture methods [17–20], which allow for a more comprehensive exposure assessment [21].

Numerous studies have reported significantly higher OCP exposures among Latinos, African Americans, and low socioeconomic status individuals, possibly due to unhealthy environments and behaviors, which might contribute to unequal rates of DM [22]. In this context, the Northern region has one of the highest agricultural densities in Mexico, where OCP have been extensively used [23], and where DM is one of the main causes of morbidity [1]. In 2021, this region registered the highest incidence rates of DM in Mexico, with more than 200 cases per 100,000 inhabitants, that is higher than the national incidence of 183 cases per 100,000 inhabitants over 9 years of age [24]. Likewise, the prevalence of DM in Northern Mexico in 2018, was 12.8%, which exceeded the national prevalence of Mexico (10.3%) and the United States (8.2%) in the same year [1, 25]. Therefore, our objective was to evaluate the association between DM and serum concentrations of OCP in residents of that region, through individual and mixture approaches, as well as to identify those OCP with greater importance within the mixture.

## Materials and methods

### Design and study population

This report presents a secondary cross-sectional analysis conducted on the control group of an original population-based case-control study, that was performed between 2007 and 2011, to evaluate various environmental and genetic factors associated with breast cancer, in women residing in six northern Mexican states: Sonora, Chihuahua, Coahuila, Durango, Tamaulipas, and Nuevo León. We have detailed the respective methodology elsewhere [26, 27]. Briefly, we included 1,030 women with no personal history of cancer (controls), who were 18 years of age or above, and had a time of residence of at least one year in the study area. We identified controls through the master sample framework used for the National Health Survey; the participation rate was 99.9%. In these group, we identified 228 diabetic women through a structured questionnaire when they answered affirmatively to the question: Have you ever been diagnosed with diabetes? The rest 802 women were considered as non-diabetic.

The Research, Ethics and Biosafety Committees of the National Institute of Public Health evaluated and accepted the protocol (No. 187–2006), which was conducted under the principles of the Declaration of Helsinki.

### Interviews and blood samples

After signing the informed consent, trained personnel interviewed participants to obtain their sociodemographic, clinical, reproductive, dietary, and lifestyle characteristics. We obtained anthropometric measurements to estimate the body mass index. The women donated a 10 ml sample of venous blood that was centrifuged and stored at -70 °C in cryovials until analysis.

### Determination of organochlorine pesticides and metabolites

According to the protocol recommended by the US Environmental Protection Agency, we extracted OCP and metabolites from serum with hexane, and quantified them in an Agilent 7890 gas chromatograph equipped with an electron capture micro detector at the Toxicology Department in CINVESTAV [28]. Each sample was enriched with 5 ng/µl of 4,4’-dichlorobenzophenone as internal quality control, showing an average recovery of 98.6 ± 5.2%. In each batch of samples, we also included a bovine fetal serum blank (SFB, Gibco Industries Inc., Langley, E.U.A.) enriched with 20 ng/ml of a certified mixture of OCP, with recoveries ranging from 93.8 to 103.5%. Additionally, we randomly selected a sample in each batch, and analyzed it in duplicate, obtaining coefficients of variation of < 10%. Detection limits (DL) for each OCP or metabolite were as follows (ng/ml): p,p´-DDT: 0.034; o, p´-DDT: 0.333; p, p´-DDE: 0.026; o, p´-DDE: 0.026; p, p-dichlorodiphenyldichloroethane (DDD): 0.022; o, p´-DDD: 0.019; HCB: 0.012; α-HCH: 0.031; β-HCH: 0.054; γ-HCH: 0.020; trans nonachlor: 0.053; cis nonachlor: 0.038; trans chlordane: 0.039; cis chlordane: 0.048; oxychlordane: 0.072; α-endosulfan: 0.031; β-endosulfan: 0.070; endosulfan sulfate: 0.126; dieldrin: 0.127; aldrin: 0.012; endrin: 0.044; heptachlor: 0.019; heptachlor epoxide: 0.008; and mirex: 0.027. We assigned the value corresponding to the respective limit of detection divided by two, in samples with pesticide concentrations below the DL [29]. We determined serum lipid concentration for each sample by colorimetry using the 100–1270 kit from SPINREACT S.A.U. (Girona, Spain). We expressed OCP concentrations in ng/g (lipid base). Due to insufficient serum sample for laboratory analysis, the final sample size of this study was 214 diabetics and 694 non-diabetics.

### Statistical analysis

**Fig. 1:**
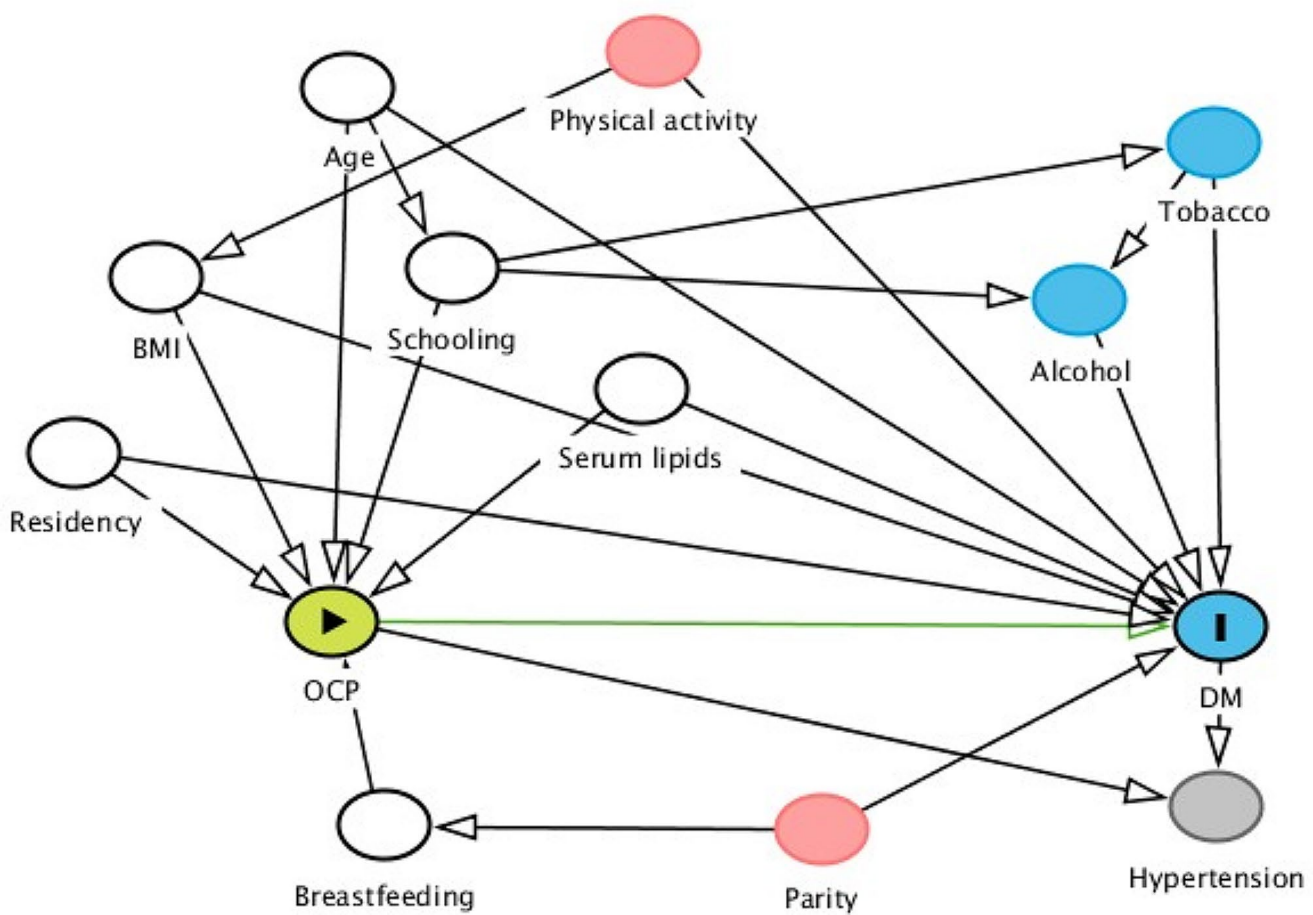
Directed acyclic graph of diabetes mellitus (DM) and organochlorine pesticides (OCP) exposure. Green line: potential causal path; white node: adjusting variables; pink node: ancestor of exposure and outcome; blue node: no-confounding variable

We evaluated the crude association between DM and selected characteristics through logistic regression models. We estimated the Spearman correlation coefficients among OCP, and used the Mann-Whitney U test to compare serum lipid-based OCP concentrations between diabetic and non-diabetic women. Based on the distribution of non-diabetic women OCP concentrations, we estimated quartiles, and evaluated their individual association with DM through adjusted logistic regression models. We selected the following minimum set of confounders by Directed Acyclic Graphs: age (years), schooling (years), body mass index (kg/m^2^), breastfeeding (months), and state of residence (Fig. 1). We also adjusted by total serum lipids (mg/dL), to reduce potential residual confounding of lipid-based OCP concentrations [30].

We used adjusted Weighted Quantile Sum (WQS) regression models with binomial specification to identify OCP of concern within mixtures, and their association with DM. With this methodology, we constructed a weighted index of OCP concentrations in quartiles, constrained to be positive and negative, with 100 bootstrap samples each [31]. We expressed the relative strength of each OCP as a percentage of the total weight index. Those exceeding 4.2% (1/24 OCP) were established as OCP of concern, indicating a greater contribution to DM than expected by chance [32]. To evaluate the stability and potential generalizability of the results, we applied a repeated holdout validation, with a cross-validation and bootstrap resampling. For this, we randomly split the data into training (40%) and validation (60%) test sets, and repeated the WQS regression 100 times to simulate a distribution of validated results from the underlying population [31]. We repeated the above analysis excluding hypertensive women. As a sensitivity analysis, we estimated the association of the mixture excluding total serum lipids in the adjustment. We expressed the results as odds ratios (OR) and their respective 95% confidence intervals (95%CI), for each quartile change in the WQS index. Likewise, we reported the average weight for each OCP within the index. We performed the analyzes in R software, version 4.3.0 (R Core Team, 2023), with the “gWQS” package [33]; and STATA 14 (StataCorp, College Station, TX, USA).

## Results

Diabetic women were older than non-diabetic women. Schooling was negatively associated with this disease, while breastfeeding, total serum lipids and hypertension were positively associated. DM was less reported among residents of Chihuahua compared to those in other states of residence. No association was observed between DM and body mass index, tobacco use nor alcohol consumption (Table 1).

**Table 1:** Association between diabetes mellitus and selected characteristics in the study sample

Characteristics	p50(p10, p90) ^a^		OR (95%CI)
	Diabetic women (*n* = 214)	Non-diabetic women (*n* = 694)	
Age (years)	59.00 (46.00, 70.00)	53.00 (37.00, 71.00)	**1.04 (1.03, 1.06)**
Schooling (years)	5.00 (0.00, 9.00)	6.00 (1.00, 12.00)	**0.93 (0.89, 0.97)**
Body mass index (kg/m^2^)	29.85 (24.34, 38.14)	29.83 (22.96, 38.58)	1.00 (0.98, 1.03**)**
Breastfeeding (months)	48.00 (18.00, 168.00)	36.00 (12.00, 156.00)	**1.01 (1.00, 1.02)** ^**b**^
Total serum lipids (mg/dl)	1052.00 (593.00, 1642.00)	871.50 (593.00, 1642.00)	**1.23 (1.16, 1.30)** ^**c**^
	**%**		
	**Diabetic women**	**Non-diabetic women**	
Hypertension			
No	45.33	78.96	1
Yes	54.67	21.04	**4.53 (3.27, 6.27)**
Tobacco consumption ^d^			
No	72.43	71.04	1
Yes	27.57	28.96	0.93 (0.66, 1.31)
Alcohol intake			
No	92.06	88.62	1
Yes	7.94	11.38	0.67 (0.39, 1.16)
Residency			
Chihuahua	7.94	15.13	1
Coahuila & Durango	32.71	31.99	**1.95 (1.09, 3.47)**
Sonora	27.10	24.64	**2.10 (1.16, 3.79)**
Nuevo León ^e^	32.24	28.24	**2.17 (1.22, 3.89)**

^a^ p10: 10th percentile; p50: 50th percentile; p90: 90th percentile.

^b^ for every three-month increase

^c^ total serum lipids as deciles

^d^ has consumed at least 100 cigarettes in their lives

^e^ 15 women from Tamaulipas

Bold numbers correspond to odds ratios whose coefficient intervals do not cross the null value = 1.

Detection of OCP concentrations in serum samples (> DL) ranged from 0.77% for β-endosulfan to 98.79% for p, p’-DDE (Table 2). The OCP were positively correlated with each other, except p, p’-DDE and HCB, which were related to each other but not with the rest of OCP (data not shown). Serum concentrations of p, p’-DDE, HCB and trans nonachlor were higher in diabetic women, while concentrations of p, p’-DDT, o,p’-DDT, p,p’-DDD, o,p’-DDD, α-HCH, γ-HCH, cis nonachlor, trans chlordane, cis chlordane, oxychlordane, α-endosulfan, β-endosulfan, endosulfan sulfate, dieldrin, aldrin, endrin, and mirex were higher in non-diabetic women. We did not observe significant differences regarding o, p´-DDE, heptachlor and heptachlor epoxide; nor significant changes when excluding hypertensive women, except for heptachlor epoxide whose concentration was higher in diabetic women (Table 2).

**Table 2:** Serum concentrations of organochlorine pesticides among diabetic and non-diabetic women

Pesticide (ng/g)	% > DL	All sample p50(p25, p75) ^a^		Excluding hypertensive women p50(p25, p75) ^a^	
		Diabetic women (*n* = 214)	Non-diabetic women (*n* = 694)	Diabetic women (*n* = 97)	Non-diabetic women (*n* = 548)
p, p’,DDT	8.70	1.75 (1.36, 2.84)	2.03 (1.61, 2.94)*	1.79 (13.8, 3.00)	2.06 (1.64, 3.06)*
o, p’,DDT	5.07	16.32 (12.77, 21.43)	19.69 (15.74, 27.66)*	16.52 (13.35, 23.19)	20.05 (16.13, 29.84)*
p, p’,DDE	98.79	6701.00 (3236.00, 15218.80)*	2917.89 (363.22, 9441.76)	5795.28 (32779.84, 12767.96)*	2231.58 (286.88, 8303.10)
o, p’,DDE	29.30	1.91 (1.20, 3.88)	1.89 (1.34, 3.79)	2.39 (1.23, 4.43)	1.94 (1.37, 3.79)
p, p’,DDD	24.12	1.35 (0.94, 2.74)	1.52 (1.11, 2.99)*	1.25 (0.92, 2.74)	1.58 (1.13, 2.99)*
o, p’,DDD	29.85	1.21 (0.82, 3.00)	1.37 (0.96, 3.01)*	1.17 (0.80, 4.41)	1.41 (0.98, 3.02)
HCB	49.45	198.29 (2.94, 370.55)*	1.67 (0.70, 230.77)	178.83 (2.60, 319.29)*	1.30 (0.69, 196.67)
α-HCH	4.19	1.51 (1.20, 1.94)	1.85 (1.49, 2.55)*	1.55 (1.26, 2.04)	1.88 (1.52, 2.72)*
β-HCH	34.47	3.71 (2.39, 254.15)	4.23 (2.91, 26.03)	3.55 (2.39, 15.49)	4.21 (2.91, 19.51)
γ-HCH	4.96	0.99 (0.77, 1.28)	1.19 (0.95, 1.64)*	1.0 (0.81, 1.31)	1.21 (0.98, 1.79)*
Trans nonachlor	41.30	6.57 (3.22, 9.12)*	4.53 (2.93, 8.19)	6.37 (3.0, 10.14)*	4.41 (2.93, 8.25)
Cis nonachlor	19.71	2.16 (1.59, 4.10)	2.50 (1.87, 4.98)*	2.37 (1.61, 5.33)	2.58 (1.90, 4.98)
Trans chlordane	15.09	1.98 (1.52, 3.24)	2.42 (1.89, 4.43)*	2.03 (1.59, 4.93)	2.48 (1.91, 4.81)*
Cis chlordane	12.56	2.44 (1.93, 4.04)	2.92 (2.33, 4.74)*	2.52 (1.97, 4.40)	2.96 (2.36, 4.84)*
Oxychlordane	8.92	3.78 (2.92, 6.18)	4.32 (3.48, 6.18)*	3.59 (2.90, 6.09)	4.37 (3.51, 6.56)*
α-Endosulfan	4.85	1.54 (1.20, 2.04)	1.85 (1.48, 2.51)*	1.56 (1.25, 2.19)	1.87 (1.51, 2.69)*
β-Endosulfan	0.77	3.35 (2.66, 4.28)	4.02(3.27, 5.35)*	3.41 (2.75, 4.46)	4.10 (3.31, 5.52)*
Endosulfan sulfate	2.64	6.04 (4.78, 7.88)	7.31 (5.93, 9.71)*	6.17 (4.93, 8.13)	7.41 (6.06, 10.19)*
Dieldrin	11.01	6.69 (5.45, 10.67)	7.70 (6.17, 11.76)*	6.38 (5.18, 10.63)	7.84 (6.24, 12.24)
Aldrin	5.18	0.61 (0.48, 0.82)	0.73 (0.59, 1.01)*	0.61 (0.49, 0.94)	0.74 (0.60, 1.07)*
Endrin	19.38	2.51 (1.78, 5.69)	2.81 (2.15, 5.52)*	2.51 (1.83, 5.65)	2.88 (2.17, 5.47)*
Heptachlor	19.38	1.15 (0.84, 2.86)	1.24 (0.96, 2.29)	1.21 (0.82, 3.62)	1.28 (0.98, 2.50)
Heptachlor epoxide	25.77	0.59 (0.39, 1.20)	0.54 (0.40, 1.09)	0.73 (0.41, 1.51)*	0.55 (0.41, 1.07)
Mirex	6.39	1.32 (1.04, 1.76)	1.60 (1.27, 2.30)*	1.32 (1.05, 1.75)	1.63 (1.30, 2.56)*

^a^ p25: 25th percentile; p50: 50th percentile; p75: 75th percentile

* <0.05 Mann, Whitney U test

In the adjusted models we estimated positive associations between DM and individual serum concentrations of p, p´-DDT, p,p’-DDE, o,p-DDE, HCB, trans nonachlor, oxychlordane, endrin, heptachlor and heptachlor epoxide, respectively. With the exception of oxychlordane, endrin, and heptachlor, these associations remained positive and significant when hypertensive women were excluded from the analysis (Table 3). In addition, p,p’-DDE remained statistically significant when including samples with levels > LD in the analysis: OR = 1.70 95%CI: 1.39, 2.08. Also, o,p’-DDE, HCB, Trans nonachlor, Endrin, Heptachlor, and Heptachlor epoxide maintained their positive association but lost their statistically significance, and p, p’-DDT and oxychlordane did not remained associated with DM (data not shown).

**Table 3:** Association between serum concentrations of organochlorine pesticides and diabetes mellitus

Pesticide (ng/g)	Diabetes mellitus OR (95%CI) ^a^	
	All sample (214 cases/694 control)	Excluding hypertensive women (97 cases/548 control)
p, p’,DDT	**1.25 (1.04, 1.52)**	**1.42 (1.08, 1.85)**
o, p’,DDT	0.97 (0.79, 1.20)	1.10 (0.83, 1.46)
p, p’,DDE	**1.75 (1.43, 2.14)**	**1.70 (1.28, 2.45)**
o, p’,DDE	**1.28 (1.09, 1.50)**	**1.43 (1.13, 1.81)**
p, p’,DDD	1.08 (0.92, 1.27)	1.03 (0.81, 1.31)
o, p’,DDD	1.05 (0.90, 1.22)	1.03 (0.83, 1.28)
HCB	**1.59 (1.34, 1.89)**	**1.76 (1.36, 2.28)**
α-HCH	0.87 (0.70, 1.09)	1.04 (0.77, 1.40)
β-HCH	0.98 (0.85, 1.13)	0.95 (0.77, 1.17)
γ-HCH	0.94 (0.78, 1.17)	0.99 (0.74, 1.32)
Trans nonachlor	**1.52 (1.29, 1.81)**	**1.57 (1.24, 1.98)**
Cis nonachlor	1.06 (0.90, 1.26)	1.17 (0.93, 1.48)
Trans chlordane	0.96 (0.81, 1.15)	1.06 (0.84, 1.35)
Cis chlordane	1.11 (0.92, 1.34)	1.26 (0.96, 1.64)
Oxychlordane	**1.23 (1.03, 1.48)**	1.18 (0.90, 1.54)
α-Endosulfan	1.00 (0.81, 1.23)	1.07 (0.79, 1.45)
β-Endosulfan	0.90 (0.71, 1.15)	0.96 (0.67, 1.34)
Endosulfan sulfate	0.89 (0.71, 1.12)	1.02 (0.74, 1.39)
Dieldrin	1.15 (0.96, 1.38)	1.01 (0.78, 1.32)
Aldrin	1.04 (0.85, 1.28)	1.11 (0.84, 1.47)
Endrin	**1.20 (1.01, 1.43)**	1.13 (0.89, 1.45)
Heptachlor	**1.19 (1.02, 1.39)**	1.19 (0.96, 1.48)
Heptachlor epoxide	**1.27 (1.08, 1.50)**	**1.47 (1.17, 1.86)**
Mirex	1.03 (0.84, 1.26)	0.98 (0.72, 1.30)

^b^ Adjusted by age, schooling, body mass index, breastfeeding, residency and total serum lipids; Organochlorine pesticides were included as quartiles.

Bold numbers correspond to odds ratios whose coefficient intervals do not cross the null value = 1.

**Fig. 2:**
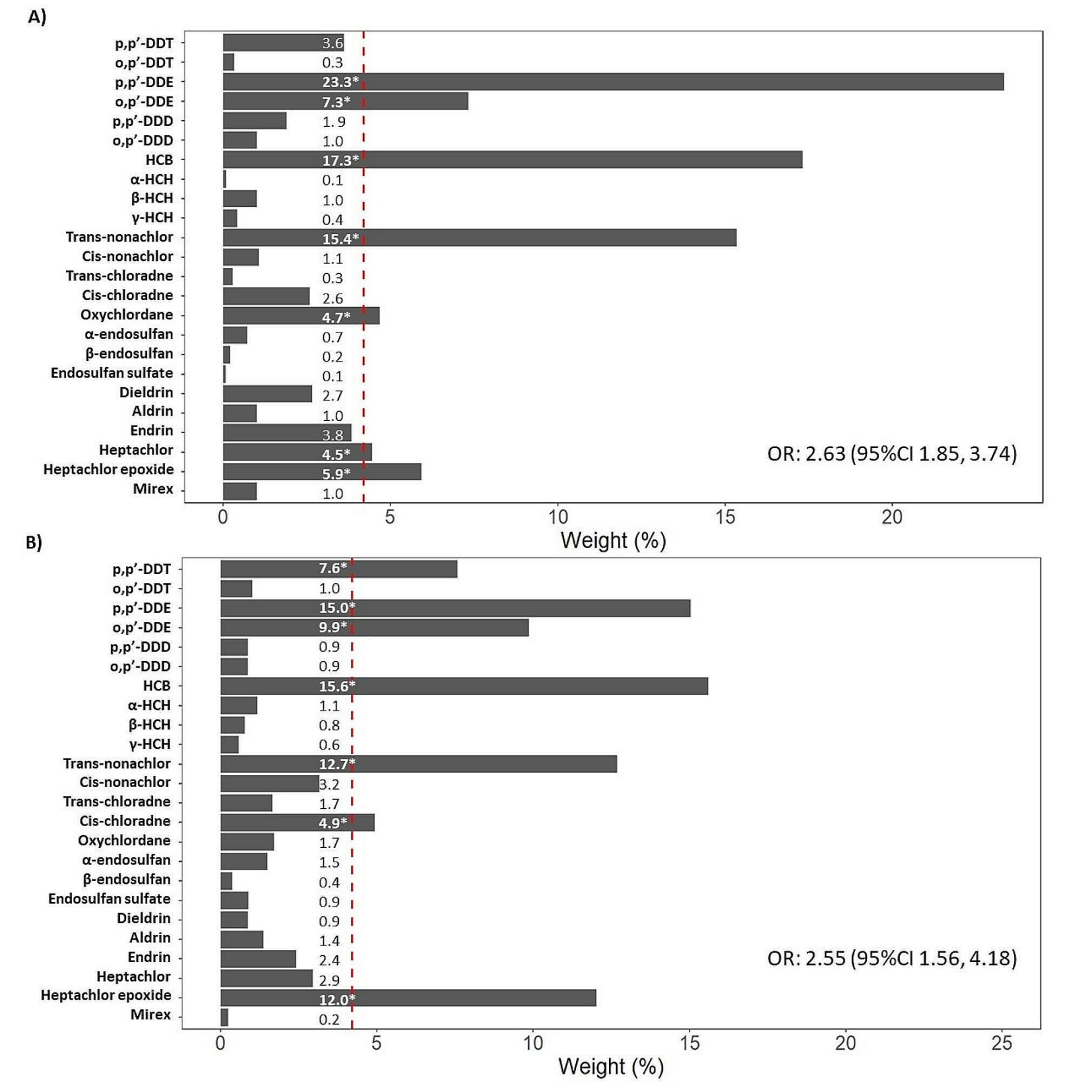
Association between diabetes mellitus and serum organochlorine pesticides mixture; (**a**) all sample, (**b**) excluding hypertensive women Note: In the bottom right corner of the graphs, we show the positive association [OR (odds ratio) and 95%CI (95% confidence interval)] between the serum concentration within the weighted index of organochlorine pesticides (OCP) and diabetes mellitus, adjusted for age, schooling, body mass index, breastfeeding, residency, and total serum lipids. Organochlorine pesticides were included as quartiles. Horizontal bars show the percentage contribution of each pesticide to the respective weighted index. Those pesticides that exceed the threshold (red dotted lines = 4.2% = 1/n OCP within the mixture) are identified as the compounds of concern within the mixture (*)

We identified a positively associated mixture of OCP with DM (OR 2.63; 95% CI 1.85, 3.74), in which p, p’-DDE (mean weight 23.3%), HCB (mean weight 17.3%), trans nonachlor (mean weight 15.4%), o, p’-DDE (mean weight 7.3%), heptachlor epoxide (mean weight 5.9%), oxychlordane (mean weight 4.7%), and heptachlor (mean weight 4.5%) stood out in their contribution. This association remained when excluding hypertensive women from the analysis (OR 2.55; 95% CI 1.56, 4.18). However, we observed a reduction in the contribution of p, p’-DDE (mean weight 15.0%); an increase in heptachlor epoxide (mean weight 12.0%); and p, p’-DDT (mean weight 7.6%) and cis chlordane (mean weight 4.9%), exhibited a contribution of concern (Fig. 2). In the sensitivity analysis, the positive association of OCP mixture with DM remained (OR 1.95; 95% CI 1.60, 2.36) after excluding total serum lipids in the adjustment. We did not find a mixture negatively associated with DM (data not shown).

## Discusión

Our results show a positive association between the prevalence of DM and an OCP mixture whose main contribution arose from p, p’-DDE, o,p’-DDE, HCB, trans nonachlor, oxychlordane, heptachlor, and heptachlor epoxide. This mixture remained associated with DM in women without hypertension, where additionally, p,p’-DDT and cis chlordane emerged as compounds of concern.

Current evidence on co-exposure to OCP and its association with DM is limited to previous studies conducted in China [17, 18, 20] and in the United States [19], in which positive associations between exposure to mixtures of OCP and DM were consistently reported. It is important to highlight that the OCP contained in the mixtures had some differences that might be explained, mainly, by regional divergences in the production, use, and restriction of these compounds, as well as their half-life in the environment. For example, in the United States, the commercial use of p, p’-DDT, which persists in the environment for more than 10 years [34], was banned in 1972 [35], while in China and Mexico it was restricted until 1983 [36] and 1991 [37], respectively. Furthermore, in these latter countries, there is the possibility of more recent exposures, related to the use of this pesticide in health campaigns to control vector-borne diseases [38]. The above might explain why this compound is present in serum samples from China [18] and Mexico that were obtained in the first decade of the 21st century, and have not been reported in the study from the United States [19] and in the most recent ones from China [17, 20]. In contrast, p,p’-DDE, whose half-life exceeds 20 years [34, 39], was found in the serum mixtures of all of them.

In our mixture, the presence of chlordane and related products such as trans nonachlor, oxychlordane, heptachlor, and heptachlor epoxide stood out, since it was recently banned in Mexico (2016) [37]. In the United States study [19] the presence of trans nonachlor and oxychlordane was identified 17 years after their ban [35], which is consistent with the at least 20 years half-life of these compounds [39]. In China, the ban on chlordane was in 2005 [36], so that in two studies the presence of its metabolites heptachlor and heptachlor epoxide was identified [17, 18]. On the other hand, HCB was identified in the samples of our study, as well as in those from the United States [19] and from China [17, 18]. Despite HCB has not been used commercially in China [36], and was banned in the United States in 1978 [35] and restricted in Mexico in 1991 [37], this compound is a byproduct in the manufacture of other chlorinated chemicals and pesticides that are still in circulation, which would explain its presence in biological samples [40].

The identification of OCP of concern within the various mixtures associated with DM partially coincides among studies. The above might be explained not only by differences in the number and concentration of the OCP evaluated, but also by their individual diabetogenic capacity and/or possible interactions among them. Three of the previous studies that evaluated exposure to OCP mixtures agreed with our results by identifying p, p’-DDE as a compound of concern positively associated with DM [17, 19, 20]; which confirms the previous findings of several studies that had evaluated it individually [13]. Additionally, we agree with the study carried out in the United States on the importance of trans nonachlor in mixtures [19]. Particularly, only studies conducted in China identified β-HCH as compound of concern [17, 18, 20]. Individually, it has been observed that some OCP have diabetogenic effects at high doses to which humans would not normally be exposed [41]. More recently, it has been shown that OCP mixtures at low doses have diabetogenic effects through one or several mechanisms such as: the production of reactive oxygen species, mitochondrial dysfunction, insulin resistance, inhibition of insulin secretion, and alterations in lipid metabolism [42–45]. However, more information is required on the possible interactions between the underlying biological mechanisms of DM due to concomitant exposure to low doses of OCP.

The results of this study should be interpreted considering some methodological aspects. Exposure to OCP has been associated with both DM and hypertension [13], which are closely related to each other [46]. Therefore, we evaluated the association of DM with the OCP mixture, excluding hypertensive women (29%), and the results remained. This suggests that OCP exposure might be associated through independent pathways with both DM and hypertension. Likewise, since serum lipids were positively associated with DM, and OCP concentrations are reported on fat basis (standardization), we also adjusted the models for serum lipids and the results remained. Additionally, although diabetic women presented higher concentrations of serum lipids, which would result in lower standardized concentrations of OCP, we did not obtain a negatively associated mixture. The above suggests that the association of DM and OCP exposure is independent of serum lipid concentrations.

It is possible that the associations reported in this study are underestimated due to the presence of non-differential measurement error in both, the DM diagnosis and OCP concentrations. On the one hand, due to self-reporting of DM and the impossibility of verifying the clinical diagnosis, it is possible that some diabetic women were not included in the group of cases. However, the prevalence of DM in this study (24%) was similar to that reported in the National Health Survey of Mexico in the same sex, age group and study period [47]. On the other hand, we had to impute a considerable proportion of OCP values because they were below the DL. However, we observed that there appears to be no relationship between the proportion of OCP concentrations below the detection limit and their identification as compounds of concern in mixtures positively associated with DM. For example, oxychlordane had more than 90% of imputed values, in contrast to p, p’-DDE which had less than 2%, and both contributed significantly to the mixture associated with DM in our study.

Although it is possible that DM might alter the metabolism and excretion of OCP [48], we do not have information in this regard, so reverse causality should not be ruled out in this study. However, several prospective studies have shown positive associations between DM both, with OCP individually and in mixtures [13, 18, 19].

## Conclusion

Despite regional differences in the composition of OCP mixtures, there is consistency regarding the contribution of p, p-DDE to DM. Because across studies there are differences in the number of OCP analyzed, that might be highly correlated with other unmeasured diabetogenic environmental contaminants, it is necessary to confirm in future studies whether the mixture we reported that includes p, p-DDE along with HCB, nonachlor, oxychlordane, heptachlor and heptachlor epoxide contribute to the development of DM. This report is the first from Latin America that provides evidence of association between exposure to OCP mixtures and the prevalence of DM.

## Data Availability

Data sharing is not possible due to ethical considerations.

## Abbreviations

DDD: Dichlorodiphenyldichloroethane
DDE: Dichlorodiphenyldichloroethylene
DL: Detection limit
DM: Diabetes mellitus
DDT: Dichlorodiphenyltrichloroethane
HCB: Hexachlorobenzene
HCH: Hexachlorocyclohexane
OCP: Organochlorine pesticides
WQS: Weighted Quantile Sum
